# A Quantitative Ethnopharmacological Documentation of Natural Pharmacological Agents Used by Pediatric Patients in Mauritius

**DOI:** 10.1155/2014/136757

**Published:** 2014-05-19

**Authors:** M. Fawzi Mahomoodally, D. Priyamka Sreekeesoon

**Affiliations:** Department of Health Sciences, Faculty of Science, University of Mauritius, 230 Réduit, Mauritius

## Abstract

The pediatric population constitutes the most vulnerable patients due to a dearth of approved drugs. Consequently, there is a pressing need to probe novel natural pharmacological agents in an endeavour to develop new drugs to address pediatric illnesses. To date, no studies have explored the use of natural therapies for pediatric health care in Mauritius. Parents (*n* = 325) from different regions of the island were interviewed. Quantitative indexes such as fidelity level (FL), informant consensus factor (*F*_IC_), and use-value (UV) were calculated. Thirty-two plants were reported to be used by pediatric patients. Gastrointestinal disorders (*F*_IC_ = 0.97) encompassing regurgitation, infantile colic, and stomach aches were the most common ailments managed with herbs. *Matricaria chamomilla *used for infantile colic and its pharmacological properties has previously been documented for pediatric patients. Product from *A. mellifera* (UV = 0.75) was the most utilized zootherapy for managing cough. Most plants and animal products reported in this study have bioactive constituents supported by existing scientific literature but their use for the pediatric population is scant. The present ethnopharmacological study has opened new perspectives for further research into their pharmacology, which can subsequently support and facilitate timely pediatric medicinal product development.

## 1. Introduction

Many children suffer from diseases that go on to cause extensive mortalities. The WHO (2012) estimated that close to 7 million children under the age of 5 died in 2011 mostly because they did not have access to simple and affordable therapies. Infectious diseases most commonly pneumonia, diarrhoea, and malaria are the main cause of deaths in children younger than 5 years [1, 2]. Caregivers frequently treat their children using natural therapies at home which are justified for various reasons [3–5]. One, natural remedies are easy to access and are also considered effective and safe to use. Second, some caregivers fear adverse effects from conventional drugs. Three, some health conditions including allergies and skin disorders, respiratory problems, and behavioural disorders are challenging to treat conventionally [6–8]. Lastly, there is a lack of approved formulations and drugs for use in the pediatric population because the development of new pediatric drugs is complex and subjected to technological, financial, and ethical challenges [9–11]. As a result, the trend is towards increased use of natural therapies in the pediatric population [7] as supported by the growing number of publications in this respect [4, 5, 10, 12–15].

Nonetheless, in spite of the many studies on the use of natural therapies for all age groups around the globe, none has documented and explored its use for pediatric healthcare in the tropical island of Mauritius. Mauritius possesses a rich biological and cultural diversity which translates into a wealth of traditional knowledge and practices including the use of natural resources for medicinal purposes by various ethnic groups. The island of Mauritius is a developing country with a rich medicinal flora and fauna and the use of traditional medicine is prevalent among the population. While foreign tourists who visit Mauritius see an idyllic, sun-soaked paradise, poverty still prevails and many Mauritians tend to prefer natural therapies for their primary healthcare [16, 17]. Indeed, traditional medicine is omnipresent in the Mauritian community whereby Mauritians still use traditional medicine for the treatment and/or management of various ailments. Nonetheless, with globalization and access to conventional medicines, Mauritians, particularly the younger people, tend to remember their use in the past as such knowledge has not been documented. Therefore, there is a pressing need to record updated primary scientific information on the different plant and animal-based therapies used by Mauritians [17].

This study therefore sets out to investigate and document primary data on herbal and animal-based therapies used by Mauritians for pediatric healthcare. Interestingly, for the first time, a quantitative survey has been designed to collect primary data for natural therapies used for pediatric care in Mauritius. It is believed that the present documentation will serve as a repertoire to record this vanishing knowledge before it is eroded completely from the island and to the scientific community. It is also anticipated that the present documentation will be fundamental to protect traditional knowledge and for the conservation of the rich biodiversity of Mauritius for future generations and to ensure Mauritius's sovereign rights over its genetic resources and utilization by first documenting them. Specifically, this paper reports a consensus on medicinal plants and animals commonly used, diversity of such therapies used, and the methods of preparation and application of such natural pharmacological agents for child care.

## 2. Methods

### 2.1. Background of Study Area

**Figure 1:**
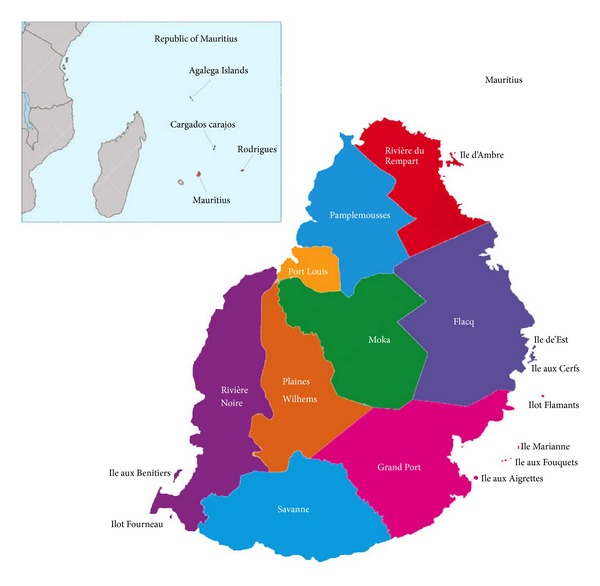
Map of Mauritius indicating the study area (spread over 9 main districts).

Mauritius is a small volcanic island, 61 km long and 47 km wide, located in the Indian Ocean, 800 km East of Madagascar [54] (Figure 1). The island has a total surface area of 1,865 km^2^ with 330 kms of coastline almost entirely surrounded by coral reefs and with an estimated population of 1,299,000 [55]. The topography of the island rises to its highest point, the Piton de la Riviere Noire, at 828 m. The geography of the land and rain distribution ensures a diverse microclimatic regime throughout the island and hence had a direct impact on both the endemic and exotic vegetation. Consequently, the flora and fauna of Mauritius are characterized by a significant percentage of endemic and indigenous species given its long geographical isolation and evolution [55].

Mauritius became an independent state in 1968 after a long history of colonialism. It was first colonized by the French from 1715 and was then by the British from 1815. The successive waves of immigration consisting of African slaves, white settlers, Indian indentured laborers, and Muslim and Chinese traders, all from different continents, contributed not only to substantial demographic changes but also to the island's ethnic composition, turning it into a multicultural society [56]. The main ethnic groups are the Bhojpuri-speaking Hindus, constituting 40.2% of the total population. The Tamils are the second largest ethnic community (13.9%), while Telugus (5.6%) and Marathis (4%) represent smaller minorities within the overall Hindu population. The Hindus have a common language (Bhojpuri), the same regional origin (Uttar Pradesh and Bihar), religious practices, and rituals [56, 57]. Previously the economy of the country was based on agriculture. However, recently the economy has successfully been diversified into textiles, tourism, banking, and business outsourcing. Today, the tourism industry in Mauritius is much more lucrative than the sugar industry, and it is also noted that there is an increase in ecotourism and geotourism in Mauritius [58, 59].

The use of traditional medicine is widespread in Mauritius and is comprised of many different forms that include Ayurveda, homoeopathy, traditional Chinese medicine, folk herbalists, traditional midwives, and many other types [16, 17]. Children are still treated using natural products especially for common ailments such as vomiting and gastrointestinal disorders.

### 2.2. Data Collection

The project was approved by the Department of Health Sciences, Faculty of Science, University of Mauritius, Mauritius. A parent survey was developed as reported in previous studies [5, 60]. The study population encompassed current natural therapies used by parents whose children fell in the age category <15 years [8, 61]. A target sample of 385 participants was interviewed during 2011–2013. This sample size was large enough at 95% confidence interval and a power of 80% with a detectable relative risk of 2 provided that the determinants of using natural therapies within the study population are not uncommon (less than 20%) or very common (greater than 80%). The sample size assumed that at least a quarter of the respondents use natural therapies. The sample size was determined using statistical of EPIINFO version 6 (http://www.cdc.gov/epiinfo CDC, Georgia) for cross-sectional studies.

Proper data was partly collected using the participatory rural appraisal method, as the key informants also became investigators themselves, participating in interviews, informal meetings, open and group discussions, and overt observations with semistructured questionnaire. The content of the semistructured questionnaire was composed of diverse information, including local names of remedies, plant or animal parts used, ailments, method(s) of preparation and administration, side-effects, and dosage [17, 62].

The interview was performed in vernacular and native languages (“Hindi,” “Bhojpuri,” and “Creole”). The questionnaire developed was strictly confidential and noncompulsory. An information sheet and a consent form were also included in order to inform the participants of the nature, implications, and objectives of this study.

During field visits, when a remedy was mentioned by the participant, where possible, the participant was encouraged to show a sample of the remedy which was collected and/or photographed. The collected sample was then identified with local botanists and experts. The data obtained during the survey was cross-checked (local names/scientific names) according to locally published books [63, 64]. Scientific names of plant species were identified according to the International Plant Name Index (IPNI: http://www.ipni.org/). A local database was constructed whereby plant samples were assigned a collection number for future reference.

The questionnaire comprised of structured open-ended and close-ended questions and family demographics such as age, gender, ethnicity, educational level, and area of residence; monthly household income and number of children were included. Parents were also asked information about their children conventional medicine use and questions concerning management of child health conditions were asked during interviews. Voluntary assistance from a local medical doctor was sought to confirm medical conditions and to establish comparisons between the local/vernacular descriptions and standard medical terms. Parents were approached randomly, particularly when they came for vaccination, dentist appointments, and routine follow-ups for their children. They were assured that the information given during the interview would be treated with utmost confidentiality and their decision to participate in the study or otherwise would not influence the treatment their child would receive.

This documentation will fully recognize the contribution of the local people who have been using the indigenous knowledge, protection of community biodiversity and intellectual property rights, and benefits, if any comes out of the study and prior informed consent for publication of the work has been obtained during the survey. Also informants were assured that this research is not for commercial purpose and aims for documentation and information dissemination on the traditional knowledge. Additionally any benefits emanating from its use must be shared according to the rules of the Convention of Biological Diversity which the government of Mauritius has ratified in 1992 [17].

### 2.3. Data Analysis

The informant or respondent consensus factor (*F*_IC_) was calculated to estimate user consensus with regard to medicinal plants. *F*_IC_ values range from 0.00 to 1.00. High *F*_IC_ values are obtained when only one or a few plant species are reported to be used by a high proportion of informants to treat a particular ailment; conversely low *F*_IC_ values indicate that informants disagree over which plant to use. The formula used is *F*_IC_ = *N*_ur_ − *N*_*t*_/(*N*_ur_ − 1), where *N*_ur_ is the number of individual plant use reports for a particular illness category and *N*_*t*_ is the total number of species used by all informants for this illness category [65].

The fidelity level (FL), the percentage of informants claiming the use of a certain plant for the same major purpose, was calculated to determine the most important species for the most frequently reported diseases or ailments as follows: FL  (%) = *N*_*p*_/*N* × 100, where *N*_*p*_ is the number of informants that claim a use of a plant species to treat a particular disease and *N* is the number of informants that use the plants as a medicine to treat any given disease [65]. The variety of use was also assessed which indicate the number of different diseases that are treated with a particular species. Use value (UV) was used to establish the relative importance of species known for plants as well as animals. It was calculated as UV = ∑*U*/*N*, where *U*  is the number of citations per species and *N*  is the number of informants.

## 3. Results 

### 3.1. Demographic Characteristics

This study was conducted to document herbal and animal-based therapies used to treat and/or manage childhood ailments and health conditions in the tropical island of Mauritius. The respondents were aged 20 years and above (Table 1). The informants were predominantly women (88%) and distributed into Hindus (40.0%), Christians (27.4%), and Muslims (20.6%) faith denominations. Respondent's average monthly income ranged between 20 001 and 30 000 Mauritian rupees (1 Mauritian rupees ~$30). Most participants had acquired at least secondary level (55%) education. The mean number of children from informants was two. An impressive 98% of users have claimed experiencing no side-effects with natural therapies (data not represented graphically).

**Figure 2:**
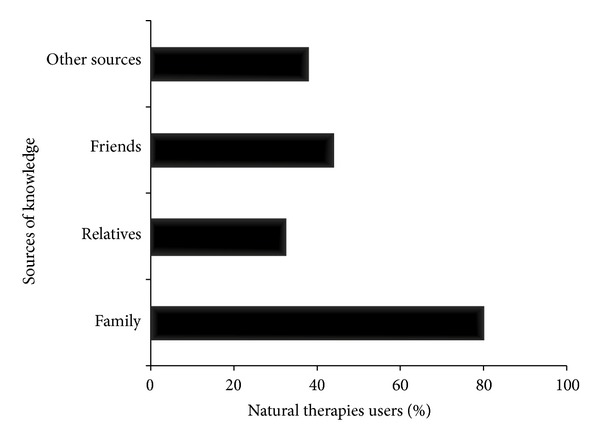
Sources of knowledge among users

The majority of informants (77.3%) stated that the natural therapies they have used are helpful, whereas 20% confirmed that such therapies were very important (data not represented graphically). Among users, the most common source of knowledge was family (80.0%). Interestingly, 31.5% and 44.1% of natural therapies users stated that they had been advised by relative and friends, respectively (Figure 2).

### 3.2. Medicinal Plants Used for Childhood Ailments and Conditions

Thirty-two medicinal plant species (distributed in 22 different families) have been documented to be in use by Mauritians for pediatric healthcare (Table 2). Medicinal plants were most commonly used to treat vomiting, infantile colic, diarrhoea, cough and flu, constipation, and chickenpox. The common methods for herbal preparation, modes of administration, and the parts of plant used have also been documented and presented in Table 2. Leaves are the most popular plant parts used and decoction is the common method of herbal preparation. Different methods of preparations were used for herbs but infusion is the most preferred method for oral preparations in children.

The categories of the plants with high number of uses mentioned for one purpose based on AbouZid and Mohamed (2011) study [66] were also compared with their fidelity level (FL) and summarized in Table 2. The most important species based on the FL scores were* Ayapana triplinervis*,* Matricaria chamomilla*,* Plectranthus madagascariensis*,* Maranta arundinacea*,* Psidium guajava*, and* Punica granatum*.

Plants with the highest number of uses mentioned for all categories were* Ayapana triplinervis* with 140 mentions for vomiting (FL = 66.9),* Matricaria chamomilla *with 99 citations for infantile colic (FL = 100), and* Plectranthus madagascariensis *with 82 mentions for cough and flu (FL = 84.5). The infusion of leaves of* Ayapana triplinervis* flavoured with a tint of sugar is the most preferred method of preparation for children. It was also reported that decoction of* A. triplinervis* was used to prepare milk for feeding the newborns. Decoction of* Bidens pilosa* (FL = 75) flowers,* Maranta arundinacea* (FL = 100) oral preparation, leaves infusion of* Polygonum poiretii* (FL = 100), decoction of* Psidium guajava *(FL = 100) leaves, and bark or fruits and decoction of rind of* Punica granatum* (FL = 100) have been reported as effective remedies against diarrhoea by informants. This was strongly supported by the high FL of these plants which demonstrated that respondents agreed on the use of these particular plants for diarrhoea.* Matricaria chamomilla *was the third most highly used herb for infantile colic (FL = 100) which was prepared from infusion of the plant's flowers and* Mentha piperita *had the highest number of mentions for stomach ache (FL = 71.8) in children. Cough and flu with a high *F*_IC_ value (0.97) were also prevalent ailments amongst pediatric individuals and* Cymbopogon citratus* was commonly used for cough and flu (FL = 52.3). Informants reported that honey and fresh lemon drops are added to extracts of* Zingiber officinale* to enhance the preparation so that it can be easily consumed by the child.* Plectranthus madagascariensis* (FL = 84.5) and* Piper betle* (FL = 79.6) were commonly used for cough and flu and have also been reported to be effective in alleviating asthmatic symptoms in children.* Azadirachta indica* (FL = 81.7) was reported to be helpful in curing chickenpox. Herbal bath prepared from decoction of the* A. indica *leaves was given to children followed by application of extracts of fresh turmeric (*Curcuma longa*). Moreover, Hindus do a special prayer known as “mata” in the local language to help in curing chickenpox, scabies, and measles. Other herbs reported with imperative implications for pediatric healthcare included* Ocimum tenuiflorum *prepared from infusion of the leaves which was reported for sleep disorders (FL = 71.4) and also to soothe infants particularly before going to sleep. Additionally,* Cardiospermum halicacabum *prepared from decoction of the whole plant was reported to be used against childhood eczema (FL = 76.9). High level of consensus (high *F*_IC_) for some species was observed, particularly for treating skin problems, vomiting, infantile colic, cough and flu, diarrhoea, and abdominal pain amongst others (Table 3).

The specific methods of use and the therapeutic uses of animal products documented from the present survey are summarized in Table 4. As compared to herbal therapies, the number of animal products in use for the pediatric population is less, with only 6 different remedies inventoried. Cough and flu are the most common ailments managed using animal derived products.

**Figure 3:**
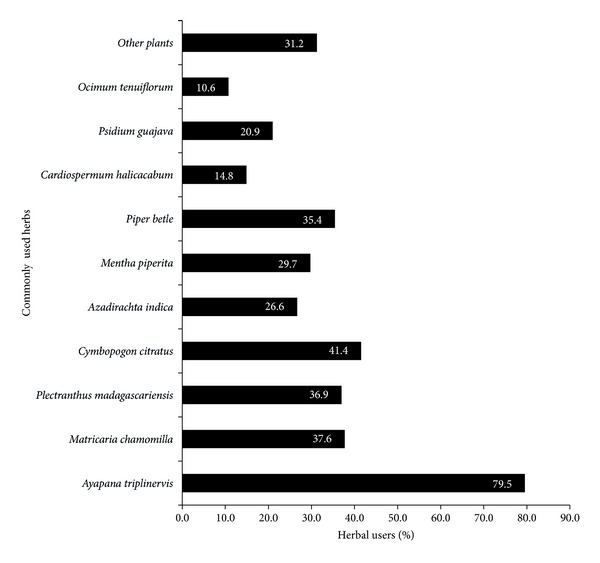
Most commonly used medicinal plants for pediatric healthcare.

As depicted in Figure 3, the most commonly used herbs are* Ayapana triplinervis* (79.5%),* Cymbopogon citratus* (41.4%),* Matricaria chamomilla* (37.6%),* Plectranthus madagascariensis* (36.9%), and* Piper betle* (35.4%).

## 4. Discussion

In spite of the extensive use of natural products with pharmacological properties from traditional medicines to treat and/or manage children ailments in developing countries like Mauritius, little has been done to document such natural pharmacological agents [67, 68]. It is generally agreed that the materials used in traditional medicines need to be carefully documented as a first step to enhance our understanding of the role of herbal and animal products in use for child healthcare and for future drug development before determining whether they are efficacious and safe to use. Although previous studies around the globe have described the use of natural therapies in pediatric patients, this study is the first to document such therapies in the tropical island of Mauritius [13–15, 60]. The main focus of this study was to document common herbal and animal therapies used to manage common childhood ailments in Mauritius.

From the present work, it was observed that Hindus and Christians were common users of natural therapies which can be explained by cultural beliefs and faith in elders' knowledge which has been bestowed upon users from previous generations (ancestors coming mainly from India and Africa). As mentioned in a previous study, therapeutic effect of natural therapies such as medicinal plants is the result of continued traditional use and this tend shows that Mauritians have left no stone unturned in exploring the biological diversity of the island [17]. It was obvious from this study that medicinal plants are the most commonly used natural product among the pediatric population [4, 5]. In a study by Ben-Arye et al. [15], it has been proposed that the use of these therapies differed by the child's age (e.g., massage in infants, prayer, or faith healing in adolescents), country of origin, and nature of the diseases. The most frequent reasons for using such therapies are based on their perceived effectiveness, due to cultural values and beliefs and fear of adverse effects from conventional drugs among other reasons [6, 7].

It was observed from the present study that various parts of plants were used for the herbal formulation, with leaves being the most frequently used plant parts. The reason for this is possibly the higher concentration of active agents in this part of the plant. This may arise from the fact that leaves act as reservoirs for photosynthesis or exudates that are thought to contain toxins for plant protection and survival which consequently find medicinal values in human health. Use of leaves for herbal remedies is generally considered to be a sustainable approach in herbal therapies as there is less risk to cause much damage and hence less threat to extinction of potential medicinal plants. In an ethnobotanical survey in Nepal, it was reported that underground plant organs were the preferred part for herbal preparations, which consequently might lead to the slow disappearance of these plants [69]. In Nigeria, herbal preparation was mostly prepared from leaves to treat measles [70]. This shows a complete agreement with data collected in the present survey and also with that of Nadembega et al. [71] where leaves were used in highest quantity. It has also been argued [72] that healers tend to use leaves due to its high availability and accessibility and being easy to identify.

This study has also provided some salient information concerning the therapeutic management of gastrointestinal disorders in children with natural therapies. The common gastrointestinal disorders reportedly managed by natural therapies were regurgitation, defecation problems, infantile colic, and frequent stomach aches.

*Ayapana triplinervis* was reported as the most common herb used with the highest number of citations and most frequently used for vomiting. A wealth of published literature is available pertaining to the pharmacological properties of this species and its use in adult population [20], though scant information is available for its use for pediatric healthcare. Extract of* A. triplinervis *has been reported to harbour panoply of bioactive molecules. For instance, a total of seven coumarins known under the trivial names ayapanin (or herniarin), ayapin, daphnetin, daphnetin dimethyl ether, daphnetin-7-methyl ether, hydrangetin, and umbelliferone have been characterized from this plant. Coumarins are considered to be components of the general defense response of plants to abiotic and biotic stresses and it has been confirmed that various substituted coumarins exhibit antimicrobial or anti-inflammatory activity and act as inhibitors of numerous enzyme systems [20]. These biological properties tend to support its use as a herbal remedy in Mauritius.

Another common medicinal plant documented in use in the present study is* Cymbopogon citratus* which was reported to be commonly used against cough and flu.* C. citratus* is known to contain various bioactive phytochemicals such as flavonoids, phenolics, terpenoids, and essential oils, which could account for its antiamoebic, antibacterial, antifilarial, antifungal, and anti-inflammatory properties amongst others [73].* Zingiber officinale* that is mixed with* C. citrates *to enhance the preparation has recently been reported to constitute mainly sesquiterpene lactones, which are responsible for its anti-inflammatory activity by inhibiting arachidonic acid metabolism and thus prostaglandin synthesis [73].

*Matricaria chamomilla *was the third most highly used herb for infantile colic. Interestingly,* M. chamomilla* is included in the pharmacopoeia of 26 countries and as a drug [74].* M. chamomilla* finds use in flatulence, colic, and hysteria, and it has also been documented to possess anti- inflammatory, antiseptic, antispasmodic, and mildly sudorific activities [74]. The dry flowers of chamomile are in great demand for use in herbal teas, baby massage oil, promoting gastric secretion, and the management of cough and cold and the use of herbal tea preparations has been reported to eliminate colic in 57% infants [74], which further endorses the plant's popularity for use against colitis in Mauritius. Indeed, active substances of chamomile have been efficient for infant colic [75]. It contains a large group of therapeutically interesting and active compound classes. Sesquiterpenes, flavonoids, coumarins, and polyacetylenes are considered the most important constituents of the chamomile drug [74, 75].

Another plant recorded in the present that has been studied extensively is* Azadirachta indica. A. indica *was reported to be helpful in curing chickenpox. It has been reported [76] that* A. indica* possesses panoply of activities including antidermatophytic and antiviral properties.* Ocimum tenuiflorum* commonly known as holy basil was used for sleep disorders and also to soothe infants particularly before going to sleep [31]. It has been shown that* Ocimum tenuiflorum* contains a high concentration of eugenol (1-hydroxy-2-methoxy-4-allylbenzene) that may be a COX-2 inhibitor, similar to modern day pain-killers and hence could possibly account for its soothing effect in children [31].* Cardiospermum halicacabum *was used against childhood eczema [77]. This species has been found to contain phytochemical constituents such as sterols, tannins, flavonoids, and triterpenes which can account for its anti-inflammatory properties [77].

Zootherapeutic practices represent an alternative to allopathic medicine in the traditional medicine of the local population [78]. Although this knowledge is gradually disappearing with time due to phenomenon like urbanization and modernization, the easy availability and accessibility and limited side-effects of these animal-based therapies are responsible for their continued popularity in the study area. In the present investigation, 6 animal-based therapies have been documented to be used in child care with honey as the most used product against cough [49]. Indeed, several studies have established the beneficial effects of honey. Its antioxidant capacity has been well studied and attributed to its polyphenolic content, namely, flavonoids and phenolics [49]. Additionally, the use of honey in wound healing in medical setting has been linked to its antimicrobial potential. Other therapeutic properties of honey including its anti-inflammatory, antiulcerous, anticancerous, and antiviral effects have been identified [49].

Nonetheless, special precautions should be taken when animal tissues are used as remedies due to the possibility of transmission of serious and widespread zoonoses such as tuberculosis or rabies [79]. According to R. R. N. Alves and H. N. Alves [78], several species of monkeys have been identified as harboring infectious diseases transmissible to man with lethal consequences. Therefore, there is a pressing need for the implementation of sanitary measures to the trade of animal or their parts for medicinal purposes. Moreover, other issues that need to be addressed when using animal-based therapies are the potential interactions among various ingredients (plants and animals), the potential interactions of folk medicines with allopathic remedies, the effects of overdose, and the possibility of toxic or allergic reactions [80].

## 5. Conclusions and Recommendations

This study is the first attempt to gather primary folk knowledge on the use of plant and animal- based therapies as natural pharmacological agents for child care in Mauritius. Failure to document such knowledge can result in losses in traditional medicines and in scientific documentation of the cultural traditions of natural therapies used in the treatment of human diseases. Our present investigation revealed that the use of plant and animal-based therapies constitutes the common legacy of all Mauritians and despite the penetration of allopathic medicine, natural therapies continue to play a crucial role in the primary healthcare system of Mauritius. This study has also demonstrated that Mauritians exploits a diversity of natural therapies which is perceived by the majority of parents to be effective. Medicinal plants which form part of the Mauritian traditional medicines have been observed to be the most preferred natural products. This can be explained based on cultural, and religious beliefs and on its availability. Moreover, herbal therapies were perceived as having minimal side-effects by parents in the present study. However, pediatricians should realise the fact that such therapies are in use and the possibility of adverse effects is not to be eliminated or ignored. Therefore, clinicians are encouraged to discuss traditional therapies with parents, since this may help to minimise potential risks and to restrain parental misconceptions and doubts. To this effect, it is recommended that doctor-patient communication and education be enhanced and clinicians should be more cooperative with patients to enable the reporting of cases and should routinely ask about such use.

Despite the prevalence and the surge of use of natural therapies amongst pediatric individuals, this remains yet a theme to be fully studied and investigated for this particular group. Further research and investigations are needed to explore the potential of medicinal herbs reported herein which could be the basis of an evidence-based investigation to discover new drugs. Natural therapies with the highest number of citations warrant further clinical studies geared towards pediatric healthcare.

## Figures and Tables

**Table 1:** Demographic characteristics of informants.

Characteristic	Description	Percentage
Age categories	20–24	7.4
	25–29	15.7
	30–34	21.8
	35–39	18.8
	40–44	16.3
	>45	20.0

Gender	Male	11.4
	Female	88.6

Residence	Urban	50.2
	Rural	49.8

Ethnicity	Muslim	20.6
	Hindu	40.0
	Chinese	1.2
	Christian	27.4
	Tamil	10.8

Monthly household (Mau Rs) 1 US$ = Rs 31	<10000	23.6
	10001–15000	10.8
	15001–20000	21.2
	20001–30000	23.1
	30001–40000	12.6
	>40000	8.6

Level of education	Primary	24.6
	Secondary	55.1
	Tertiary	20.3

Number of children	1	35.1
	2	36.6
	3	17.2
	>3	11.1

**Table 2:** List of medicinal plants with the reported methods of preparation and therapeutic uses.

Family/plant species/collection number	VN/CEN	Part used	Method of preparation	Pediatric use/ailment treated (Number of citation, fidelity level)*	Recorded literature uses
**Amaranthaceae** *Chenopodium ambrosioides* L. (PSMTS09)	Bautrisse	L	Decoction of leaves is taken orally twice daily for 5 days	Intestinal worms (3, 100)	Anthelmintic agent [18]

**Asphodelaceae** *Aloe vera* (L.) Burm. f. (PSMTS01)	*Aloe vera/Aloe vera *	G	Gel is smeared on wounds (twice daily) and itchy spots for at least 5 days	Antiseptic (3, 100)	Antimicrobial, anti-inflammatory [19]

**Asteraceae** *Ayapana triplinervis* (M.Vahl) R. King and H. Robinson (PSMTS03)	Ayapana/NA	AP	Decoction of aerial roots is taken orally for 1 week (3 times per day)	Vomiting (140, 66.9), diarrhoea (6, 2.9), stomach pain (5, 2.4), colitis (58, 27.8)	Antimicrobial, anti-inflammatory [20]
*Bidens pilosa* L. (PSMTS05)	Lavilbag/NA	F/WP	Decoction of flowers is taken orally twice daily for 5–7 days	Diarrhoea (3, 75), skin infections (1, 25)	Pain, fever, angina, diabetes, edema (water retention), infections, and inflammation [21]
*Matricaria chamomilla* L. (PSMTS17)	Chamomile	F	Decoction of whole plant for 1 week (twice or thrice daily) can be extended for 2-3 weeks	Infantile colic (99, 100)	Antimicrobial and anti-inflammatory [22]
*Tagetes patula* L. (PSMTS30)	Genda	F	Infusion of flowers is taken orally once for 5–7 days	Colic (2, 100)	Antifungal [23]

**Apocynaceae** *Catharanthus roseus* (L.) G.Don (PSMTS12)	Saponaire	L	Decoction of leaves is taken orally at night for 5 days	Fever (5, 100)	Anticancer, diabetes mellitus, fever, and arrest of bleeding [24]

**Balsaminaceae** *Impatiens balsamina* L. (PSMTS15)	Belzamine	L/F	Crushed leaves applied on wounds Decoction once daily is taken orally for 5 days	Apply on wounds (3, 60), colic (2, 40)	Wounds, abscesses, Antianaphylactic, antipruritic, and antidermatitic [25] Wounds, burns, and scalds [26]

**Brassicaceae** *Nasturtium officinale* W.T. Aiton (PSMTS20)	Cresson/Watercress	L	Juice extracted from crushed leaves is taken orally daily for 1 week (twice daily) and Can mix with honey	Cough (2, 100)	Antioxidant, depurative, diuretic, expectorant, hypoglycemic, and odontalgic [27] properties

**Caricaceae** *Carica papaya* L. (PSMTS08)	Papaille/papaya	S	Infusion of seeds is taken orally for 1 week each year	Intestinal worms (3, 100)	Anthelmintic activity [28]

**Euphorbiaceae** *Claoxylon glandulosum* Boivin ex Baill. (PSMTS11)	Bois d'oiseau/NA	L	Decoction of leaves is taken (one daily) orally for 5 days	Allergy (2, 100)	NA

**Fabaceae** *Aspalathus linearis* (Burm. f.) R. Dahlgren (PSMTS02)	Rooibos/rooibos		Infusion taken twice daily for 30 days	Infant colic (2, 100)	Asthma, colic disorders, allergies, and dermatological problems [29]

**Lamiaceae** *Mentha *x* piperita* L. (PSMTS18)	La menthe/mint	L	Decoction of leaves is taken orally for 2-3 days	Stomach ache (56, 71.8), colitis (20, 25.6), constipation (2, 2.6)	Antispasmodic, antiseptic, anti-inflammatory, antibacterial, and antifungal activities [30]
*Ocimum tenuiflorum* L. (PSMTS21)	Tulsi/holy basil	L	Infusion of leaves is taken orally daily for 1 month	Sleep disorders (20, 71.4), cough (8, 28.6)	Cold, headaches, and stomach disorders [31]
*Plectranthus* *madagascariensis* (Pers.) Benth. var. madagascariensis (PSMTS23)	Baume du Perou	L	Juice extracted from crushed leaves of the plant is warmed with honey and fresh lemon twice daily for 1 week	Cough and flu (82, 84.5), bronchitis (5, 5.2), asthma (10, 10.3)	Anti-bacterial, antifungal, and antihelmintic [32]
*Rosmarinus officinalis* L. (PSMTS28)	Romarin/rosemary	L	Infusion of leaves is taken orally for 5 days	Stress (4, 100)	Anti-inflammatory effect [33]

**Lythraceae** *Punica granatum* L. (PSMTS27)	Grenade/pomegranate	RI	Decoction of rind is taken orally for 5 days	Diarrhoea (4, 100)	Antioxidant activity [34]

**Marantaceae** *Maranta arundinacea* L. (PSMTS16)	NA/arrow-root	RP	RP is grilled till a brown colour is obtained and then consumed with water for 5 days	Diarrhoea (13, 100)	Tuberculosis, weakness [35]

**Meliaceae** *Azadirachta indica* A. Juss. (PSMTS04)	Lilas de Perse/Neem	L	Herbal bath is given to child prepared from decoction of the leaves followed by application of green turmeric	Vomiting (140, 66.9), diarrhoea (6, 2.9), stomach pain (5, 2.4), colitis (58, 27.8)	Antimicrobial, anti-inflammatory [20]

**Myrtaceae** *Psidium guajava* L. (PSMTS26)	Goyave/guava	L/B	Decoction taken orally for 3 days, thrice daily	Diarrhoea (55, 100)	Antispasmodic and antimicrobial properties in the treatment of diarrhoea and dysentery and hypoglycemic agent [36]
*Syzygium aromaticum* (L.) Merr. & L.M. Perry (PSMTS29)	Ziroffe/clove	FB	Decoction is taken orally for 5 days	Cough (1, 33.3), gastrointestinal discomfort (2, 66.7)	Antimicrobial, antifungal and antiviral, anti-inflammatory, cytotoxic, and anesthetic properties [37]

**Piperaceae** *Piper betle* L. (PSMTS22)	Betel/betel	L	Juice extracted from crushed leaves of the plant is warmed with honey and fresh lemon	Cough and flu (74, 79.6), bronchitis (4, 4.3), asthma (15, 16.1)	Antioxidant, antimicrobial, antifungal, anti-inflammatory, and radio-protective properties [38]

**Poaceae** *Cymbopogon citratus* (DC. ex Nees) Stapf (PSMTS14)	Citronel/citronella	WP	Decoction of plant is taken orally for 7–10 days. *Zingiber officinale*, sugar and fresh lemon drops, is often added to enhance the preparation	Cough and flu (57, 52.3), fever (52, 47.7)	Inflammation, digestive disorders, diabetes, nervous disorders, and fever [39] Anxiolytic, hypnotic, and anticonvulsant properties [40]

**Polygonaceae** *Polygonum poiretii* (Meisn.) K.L. Wilson (PSMTS24)	Persiker	L	Infusion of leaves is taken orally for 7 days after meal	Diarrhoea (4, 100)	Antimicrobial, anti-inflammatory [41]

**Rosaceae** *Prunus persica* (L.) Batsch. (PSMTS25)	Peche/peach	L	Decoction of leaves is taken orally for 5 days twice daily	Intestinal worms (2, 100)	Antioxidant properties [42]

**Rubiaceae** *Morinda citrifolia* L. (PSMTS19)	Noni	L	Decoction of leaves is taken orally for 3 days after meal	Pain relief (1, 100)	Antioxidant properties [43]

**Rutaceae** *Citrus* × *aurantium* L. (PSMTS10)	Bigarade/citrus	FR/L	Pressed juice of the fruit is taken orally. It is flavoured with sugar. Decoction of leaves is taken orally for 2 weeks	Flu (3, 100)	Anti-inflammatory properties [44]
*Toddalia asiatica* (L.) Lam. (PSMTS31)	Patte poule/NA	L	Infusion of leaves is taken orally for 3 days	Fever (2, 100)	Fever and pulmonary infections [45]

**Sapindaceae** *Cardiospermum halicacabum* L. (PSMTS07)	Liane pok pok/NA	L	Herbal bath is given to child prepared from decoction of the leaves for 10 days	Eczema (30, 76.9), allergy (9, 23.1)	Antioxidant and anti-inflammatory [46] effects

**Solanaceae** *Brugmansia suaveolens* (Humb. and Bonpl. ex Willd.) Bercht. and C. Presl (PSMTS06)	Fleur trompette/NA	F	Infusion of flowers is taken orally for 3 days	Asthma (3, 100)	NA

**Zingiberaceae** *Curcuma longa* L. (PSMTS13)	Safran/green turmeric	R	Taken with milk orally at night for 10 days before sleep	Cough (4, 100)	Antimicrobial, antifungal, insecticidal, anti-inflammatory, and antioxidant properties [47]
*Zingiber officinale* Roscoe (PSMTS32)	Gingembre/ginger	R	Decoction used *Cymbopogon citrates* for 10 days before sleep	Cough and flu (4, 100)	Anti-inflammatory, antiemetic, chemoprotective effects [48]

Plant part used: G: gel; AP: aerial parts; L: leaves; WP: whole plant; F: flowers; S: seeds; RP: root powder; B: bark; R: rhizome; RI: rind; FR: fruit; FB: flower buds; VN/CEN: vernacular name/common English name, NA: not available. *The categories of the plants with higher number of uses mentioned (for one purpose) were also compared with their fidelity level.

**Table 3:** *F*_IC_ calculated for the most common ailments.

Diseases	*F*_IC_
Cough and flu	0.97
Gastrointestinal ailments	0.97
Fever	0.96
Skin problems	0.99

**Table 4:** Animal products and the reported therapeutic uses for pediatric health care.

SN	VN/CEN	Part(s) used and method of preparation	Therapeutic uses	UV	Reported literature uses
*Bos taurus *	Martaige vache/cow's ghee	Honey is added to cow's ghee and the mixture is warmed. Extracts of water cress are added to form a paste which is then ingested daily (before sleep) for 1 week.	Cough	0.31	NA

*Apis mellifera *	Mouche di miel/honey	Honey is mixed with pressed orange juice or boiled milk and taken orally daily for 1 week. Honey is mixed with cow's ghee as above.	Cough and flu	0.75	Antimicrobial, anti-inflammatory [49]

*Crassostrea* spp.	Zouite/oyster	Oyster with the shell is burnt for some minutes and then the inside flesh is consumed daily for 1 week.	Asthma	0.31	NA

*Donax trunculus *	Tek tek/bivalve	A soup is prepared of the bivalves. It is administered especially at night for 1 week.	Asthma	0.05	Coughs and cold for children [50], laxative, abdominal bloating pain [51]

*Helix aspersa *	Kourpa/snail	Live snails are heated and the oily substance is poured in aching ears of children for 5 days before going to bed.	Ear infection	0.25	Used for weakness [52]

*Coturnix* *coturnix japonica *	Di Zeouf caille/quail eggs	Ingestion of 1 crude egg daily for 1 month.	Asthma/respiratory diseases	0.53	Asthma [53]

SN: scientific name, VN: vernacular name, CEN: common English name, and UV: use value.
